# Prevalence and Drivers of COVID-19 Vaccine Hesitancy among Czech University Students: National Cross-Sectional Study

**DOI:** 10.3390/vaccines9090948

**Published:** 2021-08-25

**Authors:** Abanoub Riad, Andrea Pokorná, Natália Antalová, Martin Krobot, Nutsa Zviadadze, Iryna Serdiuk, Michal Koščík, Miloslav Klugar

**Affiliations:** 1 Department of Public Health, Faculty of Medicine, Masaryk University, Kamenice 5, 625 00 Brno, Czech Republic; natalia.antalova@med.muni.cz (N.A.); krobot@med.muni.cz (M.K.); nutsa.zviadadze@mail.muni.cz (N.Z.); iryna.serdiuk@mail.muni.cz (I.S.); koscik@med.muni.cz (M.K.); klugar@med.muni.cz (M.K.); 2 Czech National Centre for Evidence-Based Healthcare and Knowledge Translation (Cochrane Czech Republic, Czech EBHC: JBI Centre of Excellence, Masaryk University GRADE Centre), Institute of Biostatistics and Analyses, Faculty of Medicine, Masaryk University, Kamenice 5, 625 00 Brno, Czech Republic; apokorna@med.muni.cz; 3 Department of Nursing and Midwifery, Faculty of Medicine, Masaryk University, Kamenice 5, 625 00 Brno, Czech Republic

**Keywords:** COVID-19 vaccines, cross-sectional studies, Czech Republic, decision making, mass vaccination, university students, vaccine hesitancy

## Abstract

Background: university students are believed to retain the highest levels of health literacy. They are perceived as the opinion leaders within their communities; therefore, their health-related beliefs and attitudes are deemed important for public health campaigns. This study aimed to investigate the COVID-19 vaccine hesitancy drivers among university students in the Czech Republic. Methods: a cross-sectional study using a self-administered questionnaire was carried out in the weeks before the unrestricted vaccine deployment to Czech adults. The questionnaire had 21 multiple-choice items stratified in 4 categories; demographic characteristics, COVID-19-related anamnesis and influenza vaccine experience, attitudes towards COVID-19 vaccination, and the possible drivers of COVID-19 vaccine hesitancy suggested by the WHO-SAGE. Results: out of the 1351 included students, 66.8% were females, 84.5% were Czech nationals, and 40.6% enrolled in healthcare programs. The overall COVID-19 vaccine acceptance level was 73.3%, 19.3% of participants were vaccine-resistant, and only 7.4% were vaccine-hesitant. Trust in the pharmaceutical industry, trust in healthcare providers, and perceived knowledge sufficiency predicted higher odds of vaccine acceptance. In contrast, media and social media, personal beliefs, immunity misconception, previous COVID-19 infection, and suspicions about novel vaccines and the local availability predicted higher odds of vaccine hesitancy. Conclusions: The findings of this study predict a fair probability to achieve community immunity (herd immunity) among the target population group. The primary prevention strategies in the Czech Republic need to be culturally sensitive and inclusive for foreign nationals. As one-quarter of the participating students are dependent on vaccine safety data, this study findings support the call for independent studies evaluating the side effects of COVID-19 vaccines.

## 1. Introduction

Immunization saves millions of lives every year. It presently counts for saving 2–3 million deaths from preventable infectious diseases globally [1]. Immunization is an essential function of primary preventive care deemed easily obtainable and integrated within universal health coverage (UHC) schemes [2]. University students are supposed to retain the highest levels of health literacy, which is defined as “the degree to which individuals have the ability to find, understand, and use information and services to inform health-related decisions and actions for themselves and others”, within their local communities where they are perceived as future opinion leaders [3]. Therefore, their health-related beliefs and attitudes had been a topic of interest for epidemiology and public health researchers [4,5,6,7,8]. The health literacy of university students can be influenced by several socio-economic factors, e.g., gender, household income, field of study (as healthcare vs. non-healthcare related discipline), etc. [9,10].

The coronavirus disease (COVID-19) caused by severe acute respiratory syndrome coronavirus-2 (SARS-CoV-2) was declared a pandemic in March 2020, as a growing body of evidence had emerged about its clinical manifestations, complications, and management. COVID-19, as a syndromic disease, triggered the attention of all clinical specialties as its pulmonary symptoms were not sufficiently prognostic for its presence or severity. Therefore, extrapulmonary symptoms such as neurologic, gastrointestinal, dermatologic, and oral symptoms led to large-scale debates and required further investigation to reveal their pathophysiologic mechanisms that may help better understand the novel disease and propose effective maneuvers for its management and prevention [11,12,13,14,15,16,17,18,19,20,21,22,23,24].

During the current COVID-19 pandemic, many essential routine vaccinations had been cancelled due to the lockdown, which may lead to outbreaks of diseases that can be easily prevented by immunization. The change in vaccination coverage could influence the vaccine hesitancy levels even for longer after the pandemic. In the USA, measles vaccination rates in 2020 were lower than in 2019, especially under the age of 2; consequently, the vaccination rates became equal to the ones for children from 2 to 18, but even a 2–5% decline in vaccination will have an impact due to the schools re-opening [25,26,27].

WHO defined vaccine hesitancy as the delay in acceptance or refusal of vaccines despite the availability of vaccine services [28]. This can be caused by the spread of myths about vaccines, stating, for example, that too many vaccines can overwhelm the immune system or MMR vaccines cause autism [29]. Both of these false statements have been proven wrong by studies. The statement regarding the safety of measles-mumps-rubella (MMR) vaccines has been refuted since 1999 [30]. The study showed no rapid increase in the number of cases of autism per individual after the introduction of the vaccine [30].

Parents’ attitude towards vaccination has been investigated over the last years, showing that most questions regarding vaccination arise due to the doubts of safety and efficiency. Such controversial thoughts are usually a result of disinformation or lack of knowledge of the vaccination mechanism [29]. Doubting the efficiency of immunization also contributes to vaccine hesitation. Still, vaccine hesitancy has been recognized by the World Health Organization (WHO) as one of the 10 threats to global health since January 2019 [31]. One of the most well-known vaccines that should be done at the age of 12–15 months and 4–6 years are MMR vaccines. In 2019, there was an outbreak of measles—1282 individual cases in the USA, which is the greatest number reported in the USA since 1992 [32]. The majority of cases were among people who were not vaccinated against measles [32].

As mentioned previously, the COVID-19 disease is a new challenge due to the unknown comprehensive symptomology as well as in relation to vaccination. At the beginning of 2021, less than 1% of the Czech population were vaccinated against COVID-19. The first batches of vaccines were administered to priority groups of healthcare workers and elderly people over 65. Currently (Summer 2021), over 35% have been vaccinated, without differentiating the number of doses per person [33]. There is a lack of evidence on the levels of COVID-19 vaccine hesitancy among young adults, especially university students.

This study aims to investigate the COVID-19 vaccine hesitancy among Czech universities’ students. The primary objective is to estimate the prevalence of COVID-19 vaccine hesitancy. The secondary objective is to determine the demographic risk factors and the vaccine hesitancy drivers among the students in the Czech Republic.

## 2. Materials and Methods

### 2.1. Design

A nationwide cross-sectional survey-based study was carried out between 21 April and 15 June 2021 to evaluate the COVID-19 vaccine acceptance levels among Czech university students prior to vaccine deployment to the ≤30 year-old group [34]. The study utilized an online self-administered questionnaire (SAQ) adapted from previous studies and developed through KoBoToolbox (Harvard Humanitarian Initiative, Cambridge, MA, USA, 2021) [4,35,36,37,38].

After ethical clearance, non-random sampling with the snowballing technique was used to recruit participating students from Czech universities for this study. The questionnaire was sent to the students’ organizations, the students’ representatives of the university’s academic senates, and the students’ representatives at the Council of Higher Education Institutions [39]. The snowballing technique attracted a disproportionately higher amount of participants from healthcare-related study programs; therefore, the project investigators identified several faculties from other fields to disseminate the questionnaire among their students.

The pragmatic sample size for this study was calculated using Epi-Info^TM^ version 7.2.4 (CDC, Atlanta, GA, USA, 2020) according to the total number of Czech university students, which was 299,000 as of 31 December 2020 [40]. The sample size equation assumed 50% of outcome probability with a confidence level (CI) of 95%, an error margin (E) of 3%, and 10% of non-response rate; therefore, the required sample size of this study was 1169 students from all Czech universities.

### 2.2. Participants

The target population of this study were the university students in the Czech Republic; therefore, the inclusion criteria were:

- to be enrolled at a Czech higher education institution, either a public or a private one;
- to be enrolled as a full-time student in a degree program with Czech as the language of instructions;
- to be at least 18 years old to be entitled to give their consent for participation in the study independently;

Meanwhile, the exclusion criteria were:

- to be already vaccinated among the priority group of frontline healthcare workers as some students, especially the students of healthcare study programs, were part of the healthcare volunteer teams;
- to be already vaccinated among the priority group of social and pedagogical workers as some students were part of these groups;
- to be already vaccinated among the priority group of people with chronic diseases;

Participation in this study was entirely voluntary, and the participants were able to withdraw at any time without the need to justify their decision before the data submission. The participating students did not receive any financial reward for their participation, and they were not compensated by another means to limit the selection bias as much as possible.

### 2.3. Instrument

This study’s instrument was SAQ adapted from previously conducted studies on COVID-19 vaccine hesitancy among university students [4,36,37,38]. The content validity of the proposed instrument was assessed by a panel of experts in public health, pedagogy, and sociology. Consequently, nine students at Masaryk University were invited to answer the online SAQ twice with a minimum patency period of one week between the first and the second filling. The mean Cohen’s kappa coefficient of the test re-test was 0.83 ± 0.17 (0.52–1), indicating that the SAQ retained a perfect level of reliability, and no changes were deemed required for the proposed items [41] (Table S1).

The SAQ was composed of 21 mandatory items and 3 conditional items that were only mandatory in certain cases. All the items were multiple-choice questions (MCQ), and they were stratified into four categories. The first category was for demographic information and included gender, age, nationality, study field, university, and academic year. The second category was for COVID-19-related anamnesis and influenza vaccine-related experience. The third category had only one item: a 5-point Likert scale evaluating the students’ level of acceptance for the COVID-19 vaccine, and its answers ranged from Totally Disagree (1) to Totally Agree (5). The fourth category was for the contextual, personal and vaccine-specific drivers of vaccine hesitancy as suggested by the WHO Strategic Advisory Group of Experts on Immunization (SAGE) [42].

The contextual drivers of vaccine hesitancy included (1) media and social media, (2) personal beliefs, and (3) the pharmaceutical industry. The social drivers included (1) experience with the health system, (2) perceived knowledge, and (3) immunity misconception. The vaccine-specific drivers include (1) novel vaccines, (2) safety surveillance, and (3) local availability (Table S2).

### 2.4. Ethics

The study protocol was fully reviewed and approved by the Ethics Committee (EC) of the Faculty of Medicine, Masaryk University on 20 January 2021, with reference number 3/2021. The study was conducted according to the Declaration of Helsinki, and it was reported according to the Strengthening the Reporting of Observational Studies in Epidemiology Statement (STROBE) [43,44].

All participating students provided their digital informed consent before filling in the questionnaire. No answers were saved until the participants confirmed their willingness to send out their answers by clicking “Submit”. No personal identifying data was collected from the participants, which would not allow the retrospective identification of respondents. As Masaryk University was the data controller, the whole data processing and analysis procedure was conducted in line with the European General Data Protection Regulation (GDPR) [45].

### 2.5. Statistics

The Statistical Package for the Social Sciences (SPSS) version 27 (SPSS Inc. Chicago, IL, USA, 2020) was used to perform all the statistical tests [46]. Initially, Shapiro–Wilk test was carried out to check the normality of data with a significance level (*Sig.*) of ≤0.05. Descriptive analyses were performed for the demographic variables, COVID-19-related anamnesis, influenza vaccine-related experience, willingness to take the COVID-19 vaccine, and the drivers of COVID-19 vaccine-related attitude represented by frequencies, percentages, means, and standard deviations.

Inferential statistics were carried out to evaluate the difference in terms of anamnestic variables and vaccine-related attitudes drivers across gender, nationality and study field using Chi-squared (*χ*^2^) test. The association of the COVID-19 vaccine acceptance level and demographic and anamnestic variables was evaluated using the Mann–Whitney (*U*) test and the Kruskal–Wallis (*H*) test with a confidence level of 95% and significance value (*Sig.*) ≤0.05.

Consequently, the students were classified according to their gender as female or male (declared) or sexual and gender minority (SGM). Moreover, the healthcare students were classified according to their academic year as pre-clinical medical and bachelor degree students (PMBD) who are enrolled in their 1st, 2nd, and 3rd year versus clinical medical and masters’ degree students (CMMD) who are enrolled in their 4th, 5th, and 6th year. Regression analysis of the vaccine attitudes determinants and drivers was carried out with a significance level (Sig.) of ≤0.05. In the logistic regression, vaccine hesitancy was defined as a binary outcome (1 = Not Sure/0 = any other answer), and vaccine acceptance was defined as a binary outcome (1 = Agree or Totally Agree/0 = any other answer).

## 3. Results

### 3.1. Demographic Characteristics

A total of 1932 students filled in the questionnaire properly. Only 1351 were included in the final analyses because 581 students were excluded as they had already been vaccinated by the time they responded to the questionnaire. While 1342 (99.3%) students declared their gender either as female (66.8%) or male (32.5%), there were 9 (0.7%) students who preferred not to disclose their gender as SGM members. The median age of the participants was 22 years old, and the vast majority of them (84.5%) were Czechs, followed by Slovaks (13.8%), Russians (0.7%), and Ukrainians (0.6%).

The students from 23 Czech universities took part in this study; Masaryk University in Brno (33.5%), Charles University in Prague (20.4%), and Brno University of Technology (15.7%) were the most common universities. Across the academic year, most participants were from the first three years (62.2%), while the fewest participants were doctoral students (5.7%). 

While 549 (40.6%) participants were healthcare students (HCS), the rest (59.4%) were non-healthcare students (non-HCS), including the students of technical sciences (14.8%), social sciences (9.3%), business and economics (7.8%), and natural sciences (7.7%). Across medical and healthcare faculties, the Faculty of Medicine of Masaryk University (41.7%) was the most contributing one, followed by the 2nd Faculty of Medicine of Charles University (28.1%) and the Faculty of Medicine of Charles University in Hradec Kralove (21.3%). A total of 69.4% of the HCS were PMBD (1st–3rd Year), while 29.4% were CMMD (4th–6th Year) (Table 1).

### 3.2. Anamnestic Characteristics

In total, 400 (29.6%) students had been previously infected by COVID-19, and the differences between female (28.5%) vs. male (32.1%), Czech (30.4%) vs. non-Czech (25.4%), HCS (30.8%) vs. non-HCS (28.8%) were not statistically significant (*χ*^2^ = 1.894, 2.142, and 0.613; *Sig.* = 0.169, 0.143, and 0.434 respectively). The students of law programs were the least affected (15.4%), while the students of arts and humanities were the most affected (36.5%).

However, there was no significant difference (*χ*^2^ = 1.056; *Sig.* = 0.304) between female (33.6%) and male (32.1%) students in terms of providing care to COVID-19 patients, the Czech students (33.9%) and HCS (38.4%) were significantly (*χ*^2^ = 4.602 and 13.731; *Sig.* = 0.032 and <0.001 respectively) more engaged with providing care than non-Czech students (26.3%) and non-HCS (28.8%). Among HCS, the doctoral candidates (66.7%) were the most engaged with providing care, followed by the 4th year students (59.2%) and 5th year students (47.5%), while the least engaging were the 1st year students (25.1%). Contrarily, among non-HCS, the early years students (1st–3rd Year) were more engaged with providing care than the senior years students (4th–6th Year), 31.4% vs. 25.4%, respectively.

Having COVID-19 patients or fatalities within the students’ social circles did not significantly differ across gender, nationality, or study field. While 1335 (98.8%) of the participants knew someone infected by COVID-19, only 472 (34.9%) knew someone who died with COVID-19.

Regarding their influenza vaccine experience, 280 (20.7%) students reported that they had received the influenza vaccine in the past, with 45% of them receiving it in the last three years. Non-Czech students (31.6%) and HCS (24.2%) had a significantly higher level of influenza vaccine uptake (*χ*^2^ = 17.727 and 6.897; *Sig.* < 0.001 and =0.009 respectively) compared to Czech students (18.7%) and non-HCS (18.3%). The difference between HCS (63.6%) and non-HCS (28.1%) who received the vaccine in the last three years was statistically significant (*χ*^2^ = 35.412; *Sig.* < 0.001). While the 6th year students (35.2%) had the highest level of influenza vaccine uptake among HCS, the 2nd year students (22.9%) had the highest level of influenza vaccine uptake among non-HCS (Table 2).

### 3.3. COVID-19 Vaccine-Related Attitudes

On responding to the statement “I will take COVID-19 vaccine once it becomes available for me”, 261 (19.3%) students were found to be vaccine resistant, 100 (7.4%) vaccine hesitant, and 990 (73.3%) were vaccine accepting. A 5-point Likert scale was used to assess the acceptance level; vaccine resistance was defined by both answers “*totally disagree* = 1 point” and “*disagree* = 2 points”, vaccine hesitancy was defined by the answer “*not sure* = 3 points”, and vaccine acceptance was defined by both answers “*agree* = 4 points” and “*totally agree* = 5 points” (Table 3).

The mean acceptance levels of males and females were not significantly different; however, the only significant difference between females (8.3%) and males (5.2%) was in terms of vaccine hesitancy (*χ*^2^ = 4.103; *Sig.* = 0.043). Non-Czech students (79.9%) had significantly higher levels of vaccine acceptance (*χ*^2^ = 5.543; *Sig.* = 0.019) compared to Czech students (72.1%).

**Figure 1:**
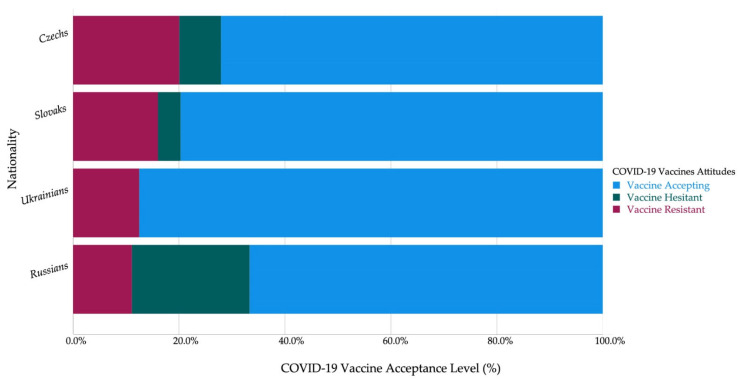
Czech university students’ COVID-19 vaccine acceptance by nationality (April–June 2021, *n* = 1346).

The non-HCS had significantly higher levels of vaccine resistance (22.6% vs. 14.6%) and vaccine hesitancy (8.7% vs. 5.5%) and a lower level of vaccine acceptance (68.7% vs. 80%) compared to HCS (*χ*^2^ = 13.371, 5.065, and 21.104; *Sig.* < 0.001, = 0.024, and <0.001, respectively) (Figure 1).

**Figure 2:**
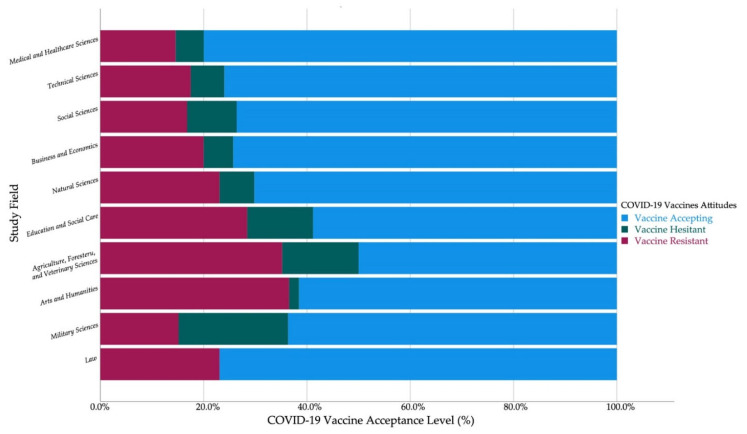
Czech university students’ COVID-19 vaccine acceptance by study field (April–June 2021, *n* = 1337).

The sixth year HCS were the most accepting of the COVID-19 vaccine (92.6%) and the first year non-HCS were the most accepting of the COVID-19 vaccine (73%). Among the non-HCS, the students of law (76.9%) had the highest level of vaccine acceptance followed by technical sciences (76%), business and economics (74.3%), social sciences (73.6%), and natural sciences (70.2%). On the other hand, the students of military sciences (21.2%) had the highest level of vaccine hesitancy followed by agriculture, forestry, and veterinary sciences (14.8%), and education and social care (12.7%) (Figure 2).

**Figure 3:**
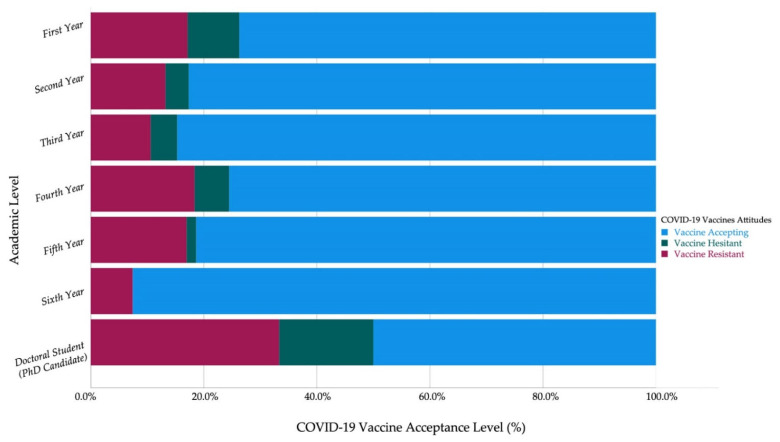
Czech healthcare students’ COVID-19 vaccine acceptance by academic year (April–June 2021, *n* = 549).

**Figure 4:**
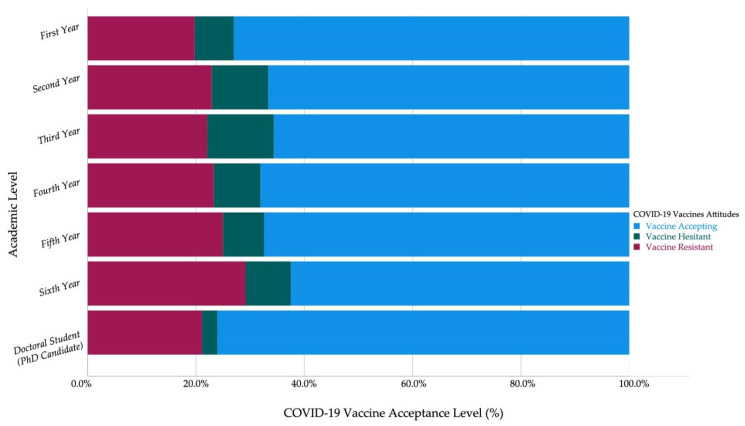
Czech non-healthcare students’ COVID-19 vaccine acceptance by academic year (April–June 2021, *n* = 788).

Among HCS, the 6th-year students (0%) and 5th year students (1.7%) had the lowest levels of vaccine hesitancy, while the first-year students (9.1%) had the highest level of vaccine hesitancy. Contrarily, the first-year non-HCS (7.2%) had the lowest level of vaccine hesitancy (Figure 3 and Figure 4).

Across medical and healthcare faculties, the students of the 2nd Faculty of Medicine of Charles University (89.6%) had the highest level of vaccine acceptance followed by the Faculty of Medicine of Charles University in Hradec Kralove (76.9%). The students of the Faculty of Pharmacy of Masaryk University exhibited the highest level of vaccine hesitancy among all medical and healthcare faculties (16%).

### 3.4. COVID-19 Vaccine-Related Attitudes Drivers

#### 3.4.1. Contextual Drivers

The vast majority of participants (81.3%) reported that mass media and social networks had no significant impact on their decision regarding COVID-19 vaccination. Females (13.6%), the students of social sciences (18.4%), and the students of education and social care (16.7%) were reportedly the most influenced by media and social media. There was no significant difference among early years students (1st–3rd Year) and their senior peers (4th–6th Year) regarding the impact of media and social media.

Similarly, the vast majority of participants (87.4%) acknowledged that their personal beliefs including cultural and religious values did not impact their vaccination decision. The non-HCS (12.2%) were more likely to reject the vaccine due to their personal beliefs than HCS (8.4%). The most susceptible non-HCS for being retained from vaccination due to personal beliefs were the students of arts and humanities (25%), followed by agriculture, forestry, and veterinary sciences (18.5%), education and social care (16.7%) and law (15.4%). The difference between early years students (1st–3rd Year) and their senior peers (4th–6th Year) was only significant among non-HCS–14.2% vs. 9.6%, respectively.

In general, most participants (74.7%) disclosed their confidence in the pharmaceutical industry to provide safe and effective vaccines. The HCS (83.2%) and non-Czech students (84.7%) had significantly (*χ*^2^ = 35.816 and 13.087; *Sig.* < 0.001 and <0.001 respectively) higher levels of confidence in pharmaceutical industry than non-HCS (68.8%) and Czech students (72.9%). Among the non-HCS, the students of arts and humanities (44.2%) had the lowest level of confidence in industry, followed by agriculture, forestry, and veterinary sciences (59.3%), education and social care (61.8%), and law (69.2%) (Table 4).

#### 3.4.2. Social Drivers

The healthcare providers were trusted by 62.4% of the participants to provide reliable information about vaccine safety. The differences between males (65.1%) vs. females (61.4%) and early years students (61.9%) vs. seniors (63.2%) were not statistically significant. The HCS (70.5%) and non-Czech students (68.4%) were more likely to trust healthcare providers than non-HCS (56.9%) and Czech students (61.3%). The students of arts and humanities (46.2%) had the lowest level of confidence in healthcare providers, followed by agriculture, forestry, and veterinary sciences (50%), social sciences (52.8%), and business and economics (53.3%).

Almost one third (30%) of the participants responded negatively to the item “do you feel you have enough information about vaccines and their safety?”. Female students (32%), Czech students (31.4%), and non-HCS (35.2%) were significantly (*χ*^2^ = 4.736, 7.479 and 25.270; *Sig.* = 0.030, 0.006 and <0.001 respectively) more likely to acknowledge that their perceived knowledge about vaccine safety was insufficient compared to male students (26.2%), non-Czech students (22%), and HCS (22.4%). Among the non-HCS, the students of agriculture, forestry, and veterinary sciences (48.1%) had the highest level of insufficient perceived knowledge, followed by military sciences (42.4%), education and social care (40.2%), and arts and humanities (34.6%).

In the HCS group, the CMMD students (72.2%) had a significantly higher level of perceived knowledge (*χ*^2^ = 4.536; *Sig.* = 0.033) compared to their PMBD colleagues (62.7%), on the other hand, the early years non-HCS (49.7%) had a similar level of perceived knowledge to their senior colleagues (53.3%). The students who trusted their healthcare providers (72.8%) had a significantly higher level of perceived knowledge (*χ*^2^ = 203.663; *Sig.* < 0.001) compared to the students who reported they did not trust healthcare providers (33.3%).

About one-quarter (24.3%) of the participants demonstrated misconception regarding the immunity system as they positively answered the item “do you believe that it is better to develop immunity by getting sick than to get a vaccine shot?”. Male students (25.3%) had a slightly higher level of immunity misconception than their female colleagues (23.8%), while Czech students (26.2%) and non-HCS (28.4%) had significantly higher levels of misconception (*χ*^2^ = 14.554 and 18.495; *Sig.* < 0.001 and <0.001 respectively) compared to non-Czech students (13.9%) and HCS (18.2%). Among non-HCS, the early years students did not have a significantly higher level of misconception, and the first-year students (33.6%) had the highest level of misconception, followed by second-year students (31.9%), and third-year students (30.1%). The students of agriculture, forestry, and veterinary sciences (51.9%) had the highest level of misconception, followed by military science (36.4%), education and social care (35.3%), and arts and humanities (32.7%).

The participants who had been infected by COVID-19 (30.3%) had a significantly higher level of misconception (*χ*^2^ = 11.023; *Sig.* = 0.001) compared to the participants who were not infected previously (21.8%). Similarly, the students with a history of influenza vaccine uptake (19.3%) had a significantly lower level of misconception (*χ*^2^ = 4.789; *Sig.* = 0.029) compared to their peers without influenza vaccine history (25.6%). Additionally, the students who reported insufficient knowledge (40.7%) were more likely to have immunity misconception (*χ*^2^ = 85.265; *Sig.* < 0.001) than their peers who thought they had sufficient knowledge (17.2%). The students who reported they trust their healthcare providers (15.6%) demonstrated a significantly lower level of misconception (*χ*^2^ = 85.894; *Sig.* = 0.001) compared to the students who did not trust their healthcare providers (45.4%) (Table 4).

#### 3.4.3. Vaccine-Specific Drivers

Regarding the vaccine-specific drivers; 21.5% of the participants thought that the novel vaccines were not trialed as rigorously as normally prescribed drugs, 25.5% of the participants agreed that their vaccination decision would be dependent on whether the side effects were being tracked or not, and 47.1% of the participants were not sure that the vaccine would be available for them when they need it.

Czech students (22.7%), non-HCS (24.1%), the students who do not trust their healthcare providers (50.9%), the students with perceived insufficient knowledge (37%), and the students suffering from immunity misconception (41.5%) had significantly higher levels of suspicion about novel vaccines (*χ*^2^ = 5.676, 7.447, 173.182, 82.196, and 101.744; *Sig.* = 0.017, 0.006, <0.001, <0.001, and <0.001 respectively) than non-Czech students (15.3%), HCS (17.9%), the students trusting their healthcare providers (14.2%), the students with perceived sufficient knowledge (14.9%), and the students without immunity misconception (15.2%).

Female students (27.7%), non-HCS (27.8%), the students who are influenced by media and social media (45.2%), the students who do not trust pharmaceutical industry (36.4%) nor their healthcare providers (39.9%), and the students reporting insufficient knowledge (39%), immunity misconception (33.8%), and suspicions about novel vaccines (36.1%) demonstrated significantly higher levels of dependence on safety surveillance data to inform their vaccination decision (*χ*^2^ = 7.542, 5.343, 39.162, 12.475, 36.536, 55.235, 15.711 and 21.692; *Sig.* = 0.006, 0.021, <0.001, <0.001, <0.001, <0.001, <0.001, and <0.001 respectively) compared to male students (20.7%), HCS (22.2%), the students not influenced by media (22.7%), the students trusting pharmaceutical industry (23.9%) and their healthcare providers (21.9%), and the students with perceived sufficient knowledge (19.8%), and without immunity misconception (22.9%), and without suspicions about novel vaccines (22.6%).

No significant differences were found between females (22.9%) vs. males (23.2%), HCS (20.9%) vs. non-HCS (24.3%), and early years students (23.9%) vs. senior years students (22.8%) in terms of their confidence of the vaccine’s availability. Non-Czech students (37.3%) had a significantly higher level of suspicion about the availability of vaccines for them locally (*χ*^2^ = 6.351, *Sig.* = 0.012) compared to Czech students (28.6%) (Table 4).

### 3.5. Distribution of COVID-19 Vaccine Acceptance Levels

The mean COVID-19 acceptance level ranged between 1 (totally disagree) and 5 (totally agree), and it did not vary significantly (*U* = 4890.5; *Sig.* = 0.283) between the students who disclosed their gender either as female or male (3.97 ± 1.37) and the SGM (3.33 ± 1.73). There was no statistically significant difference (*U* = 222175.5 and 181808.5; *Sig.* = 0.473 and 0.934) between ≤22 years-old (4.02 ± 1.32) vs. >22 years-old students (3.91 ± 1.44) nor the early years (3.98 ± 1.35) and senior years students (3.95 ± 1.41).

Czech students (3.93 ± 1.39) had a significantly lower level of acceptance (*U* = 106784.5; *Sig.* = 0.008) than non-Czech students (4.18 ± 1.29), where Slovak students (4.17 ± 1.30) and Ukrainian (4.38 ± 1.41) students had higher acceptance levels. The Technical University of Ostrava had the lowest acceptance level (3.06 ± 1.63), followed by the Czech University of Life Sciences in Prague (3.21 ± 1.55), Janáček Academy of Music in Brno (3.40 ± 1.51), and Silesian University in Opava (3.41 ± 1.47).

The medical and healthcare sciences students had the highest level of acceptance (4.21 ± 1.27) among the Czech students of all other fields. Agriculture, forestry, and veterinary sciences had the lowest acceptance level (3.20 ± 1.53), followed by arts and humanities (3.38 ± 1.64) and education and social care (3.61 ± 1.48); on the other hand, technical sciences (4.03 ± 1.36) and business and economics (3.97 ± 1.39) had the highest levels after medical and healthcare sciences. Among the HCS, the students of the 2nd Faculty of Medicine of Charles University exhibited the highest level of vaccine acceptance (4.52 ± 1.09), followed by the Faculty of Pharmacy of Masaryk University (4.36 ± 1.04), and the Faculty of Medicine of Charles University in Hradec Kralove (4.13 ± 1.25).

The first-year students (3.87 ± 1.38) had the highest level of vaccine acceptance among non-HCS, while the sixth-year students (4.54 ± 1.08) had the highest level of vaccine acceptance among HCS. Moreover, the 6th-year students had the highest level of vaccine acceptance among Czechs (4.17 ± 1.39) and non-Czechs (4.57 ± 1.09) (Table 5).

### 3.6. Time Series Analysis of COVID-19 Vaccine-Related Attitudes

The participating students’ responses had been collected between 21 April and 15 June 2021, and they were clustered into five weeks corresponding to the invitations disseminated among the target population. In the second week (28 April–4 May 2021), the highest number of responses was received (46.9%); 71.7% were females, 83.6% Czechs, and 68.4% HCS. There was a declining trend of the number of HCS over the five weeks of the study. Contrarily, there was a growing trend of the number of Czech students. (Table S3).

The time series analysis of COVID-19 vaccine-related attitudes revealed that the vaccine resistant level increased from the first week (18.7%) to the fifth week (29.5%). The vaccine accepting students fell from the first week (74.7%) to the fifth week (59%), and the mean acceptance level followed the same declining trend (Table 6).

**Figure 5:**
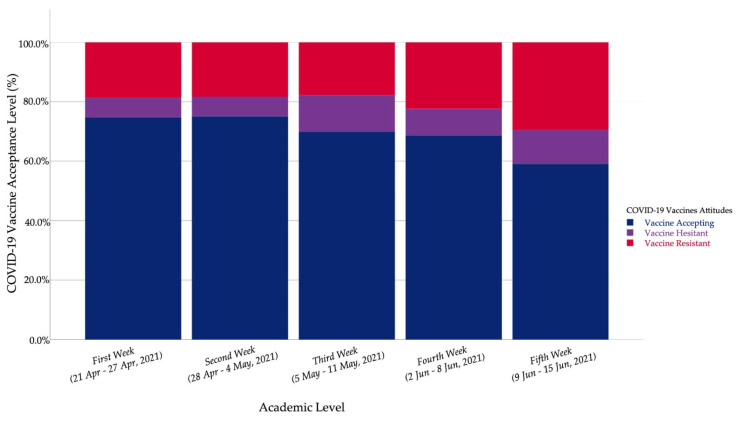
Czech university students’ COVID-19 vaccine acceptance by week (April–June 2021, *n* = 1351).

The sub-group analysis was carried out to control the effect of study field on the vaccine acceptance level. In the non-HCS group, vaccine resistance and vaccine hesitancy increased from the first week (21.8% and 7.4%) to the fifth week (29.5% and 11.5%), respectively. In the HCS group, the mean acceptance level decreased significantly (*H* = 16.121; *Sig.* < 0.001) from the first week (4.55 ± 1.05) to the third week (3.80 ± 1.37) (Figure 5).

### 3.7. Regression Analysis of COVID-19 Vaccine-Related Attitudes

The binary logistic regression of demographic variables revealed that females had an odds ratio (OR) of 1.638 times (*CI 95%:* 1.012–2.652; *Sig.* = 0.045) more than their male colleagues to be vaccine-hesitant. The SGM students also had OR of 3.627 times (*CI 95%:* 0.743–17.694; *Sig.* = 0.111) to be vaccine-hesitant more than the students who selected their gender as either female or male. The ≤22 year-old students had OR of 1.824 times (*CI 95%:* 1.185–2.808; *Sig.* = 0.006) more than the >22 year-old students to be vaccine-hesitant.

Czech students were 1.702 times (*CI 95%:* 0.871–3.329; *Sig.* = 0.120) more likely to be vaccine-hesitant than non-Czech students. HCS were 1.818 times (*CI 95%:* 1.406–2.350; *Sig.* < 0.001) more likely to accept the vaccine than non-HCS. Among the HCS, the PMBD students were 2.774 times (*CI 95%:* 0.950–8.103; *Sig.* *=* 0.062) more likely to be vaccine-hesitant than CMMD students.

The students with previous COVID-19 infection were 0.880 times (*CI 95%:* 0.678–1.143; *Sig.* = 0.338) likely to accept the vaccine, while the students who knew someone who died because of COVID-19 were 1.386 times (*CI 95%:* 1.069–1.798; *Sig.* = 0.014) more likely to accept the vaccine. The influenza vaccine increased the OR of accepting the vaccine 1.510 times (*CI 95%:* 1.099–2.067; *Sig.* = 0.011), and the recent influenza vaccine was associated with 1.157 (*CI 95%:* 0.642–2.085; *Sig.* = 0.627) odds of vaccine acceptance.

The students who depended on media and social media were 3.086 times (*CI 95%:* 1.928–4.941; *Sig.* < 0.001) more likely to be vaccine-hesitant, and the students who had no personal beliefs to retain them from vaccination were 5.036 times (*CI 95%:* 3.602–7.039; *Sig.* < 0.001) more likely to accept the vaccine. The students who trusted the pharmaceutical industry and their healthcare providers were 11.828 times (*CI 95%:* 8.886–15.742; *Sig.* < 0.001) and 5.535 times (*CI 95%:* 4.265–7.183; *Sig.* < 0.001) more likely to accept the vaccine, respectively.

The students who reported insufficient knowledge about vaccine safety were 6.061 times (*CI 95%:* 3.897–9.429; *Sig.* < 0.001) more likely to be vaccine-hesitant, and the students who had misconception about immunity were 3.347 times (*CI 95%:* 2.212–5.064; *Sig.* < 0.001) more likely to be vaccine-hesitant as well. The students who did not have suspicions about novel vaccine manufacturing were 4.876 times (*CI 95%:* 3.772–6.302; *Sig.* <0.001) more likely to accept the vaccine, while the students who depended their vaccination decision upon the safety surveillance were 2.965 times (*CI 95%:* 1.961–4.483; *Sig.* <0.001) more likely to be vaccine-hesitant. The students who were confident that they would be able to find the vaccine in their local health centers were 1.362 times (*CI 95%:* 1.009–1.838; *Sig.* = 0.044) more likely to accept the vaccine. Hosmer and Lemeshow (HL) tests indicated that the vaccine hesitancy (*χ*^2^ = 0.091; *Sig.* = 1.000) and vaccine acceptance (*χ*^2^ = 14.794; *Sig.* = 0.063) models have good fitness (Table 7).

## 4. Discussion

Overall, 73.3% of the Czech universities’ students participating in this study were willing to receive COVID-19 vaccines whenever possible (vaccine accepting group); on the other hand, 19.3% were unwilling to get vaccinated (vaccine-resistant group), and only 7.4% were hesitant about COVID-19 vaccination (vaccine-hesitant group). The current acceptance level of Czech students is higher than what was reported in Qatar (62.6%), France (58%), USA (50.6%), and Jordan (34.9%), and lower than what was reported initially in Italy (86.1%) [47,48,49,50,51].

However, our sample was not equally distributed across gender, as the majority of participating students were females (66.8%), and the latest report of the Czech Statistical Office (ČSÚ) revealed that 55.6% of public university students and 57.1% of private university students were females [52]. According to the same report, 15.6% of the students enrolled in Czech universities are non-Czechs, similar to the percentage of non-Czech students in our sample of 15.5% [52]. While 12.4% of the public university students in the Czech Republic are enrolled in medical and healthcare programs, this group was over-represented in our sample, with 40.6%, due to the recruitment strategy used in this study which relied primarily on students representatives from these programs [52].

Female students (8.3%) in our study were significantly more hesitant (*χ*^2^ = 4.103; *Sig.* = 0.043) about COVID-19 vaccination than their male colleagues (5.2%). Riad et al. 2021 found that females within a global sample of 6639 dental students from 22 countries were significantly more hesitant to receive COVID-19 vaccine (*χ*^2^ = 9.18; *Sig.* = 0.02) [4]. The same finding was reported by Patelarou et al. 2021 in seven European countries, Tavolacci et al. 2021 in France, and Sallam et al. 2021 in Jordan [48,50,53]. Interestingly, the acceptance levels were not different between our female and male students, consistent with what was reported recently among medical students in Slovakia and dental students globally [4,54].

Da Silva et al. 2021, found that mistrust of the healthcare system and vaccine-related social stigma were the main barriers for men of SGM in the USA to accept COVID-19 vaccination [55]. Only 0.7% of our participants were SGM, even though they had OR of 3.627 times (*CI 95%:* 0.743–17.694; *Sig.* = 0.111) to be vaccine-hesitant than their colleagues. This finding can be explained by the fact that SGM students in our sample had higher mistrust of the health system (44.4% vs. 19.9%) and pharmaceutical industry (44.4% vs. 12.8%) than their colleagues who identified their gender as females or males.

Non-Czech students had a substantially higher level of COVID-19 vaccine acceptance (79.9%) compared to international students in China (36.4%) where the international students were challenged by various barriers for getting vaccinated, including the potential side effects, vaccine availability, interrupting their routine activities, and insufficient knowledge about vaccine safety [56]. In Qatar, citizens were 1.68 times (*CI 95%:* 1.30–2.16; *Sig.* < 0.001) more likely to be COVID-19 vaccine-hesitant than foreign expats [57]. This trend was also found in our sample, as the Czech students were 1.702 times (*CI 95%:* 0.871–3.329; *Sig.* = 0.120) more likely to be vaccine-hesitant than non-Czech students.

On comparing the vaccine hesitancy drivers between Czech and non-Czech students, the non-Czech students had better scores for all the proposed drivers that could predict their higher level of vaccine acceptance except for the “vaccine availability” item, as the non-Czech students (37.3%) had a significantly higher level of suspicion about the availability of vaccines for them locally (*χ*^2^ = 6.351, *Sig.* = 0.012) compared to Czech students (28.6%). The Ministry of Health (MoH) of the Czech Republic issued a press release on 1 February 2021, indicating that only the foreign nationals who had access to the public health insurance system would be entitled to receive COVID-19 vaccines unrestrictedly, while the EU nationals who were entitled to essential care in their home countries were required to perform a few steps in priori for vaccine registration, and the third countries nationals were only able to get the vaccine through commercial insurance companies [58]. This nationality-based prioritization policy might have adversely impacted the non-Czech students’ confidence regarding their access to the vaccines locally.

Al-Mulla et al. 2021 found that HCS (80.6%) in Qatar had significantly higher levels of COVID-19 vaccine acceptance (*χ*^2^ = 5.40, *Sig.* = 0.02) compared to non-HCS (61%) [47]. Similarly, Polish HCS (91.99%), French HCS (50.8%), and Jordanian HCS (43.5%) were more vaccine accepting than Polish non-HCS (59.42%), French non-HCS (32.3%) and Jordanian non-HCS (24%) [37,48,50]. In our sample, non-HCS had significantly higher levels of vaccine resistance (22.6% vs. 14.6%) and vaccine hesitancy (8.7% vs. 5.5%) and a lower level of vaccine acceptance (68.7% vs. 80%) compared to HCS (*χ*^2^ = 13.371, 5.065, and 21.104; *Sig.* < 0.001, = 0.024, and <0.001, respectively).

Out of the 581 excluded students, 487 (83.8%) were HCS who were already vaccinated due to their engagement with voluntary healthcare teams. Therefore, the overall vaccine acceptance levels among HCS might have been underestimated in this study because a considerable portion of HCS were already vaccinated and excluded from the final analysis. The differences between HCS and non-HCS should not be attributed exclusively to the type of education that those students receive, as the perceived knowledge (HCS: 70.5% vs. non-HCS: 56.9%; *Sig.* < 0.001) and immunity misconception (HCS: 18.2% vs. non-HCS: 28.4%; *Sig.* < 0.001) may act as predictor variables for the vaccine acceptance, which could be mediated by other drivers including trust in the pharmaceutical industry (HCS: 83.2% vs. non-HCS: 68.8%; *Sig.* < 0.001), trust in the health system (HCS: 70.5% vs. non-HCS: 56.9%; *Sig.* < 0.001), suspicions about novel vaccines (HCS: 17.9% vs. non-HCS: 24.1%; *Sig.* = 0.006), and dependence on side effects surveillance (HCS: 22.2% vs. non-HCS: 27.8%; *Sig.* = 0.021). The significance and interactions of these complex drivers are better demonstrated by a statistical equation model (SEM) that can classify and combine all the proposed variables predicting COVID-19 vaccine acceptance, including the study field, the study program, and the academic year.

The HCS in our study demonstrated an acceptance level of 80%, which is lower than what was reported in Poland (91.99%), India (89.41%), Slovenia (82%), Italy (>80%), Portugal (>80%), USA (>80%), Canada (>80%), Indonesia (>80%), and Malaysia (>80%) by HCS [4,37,59]. The PMBD students were 2.774 times (*CI 95%:* 0.950–8.103; *Sig. =* 0.062) more likely to be vaccine-hesitant than CMMD students; this difference may support the earlier proposition that healthcare curricula may play a key role in shaping students’ beliefs and attitudes towards vaccination. The clinical dental students were more vaccine accepting than their pre-clinical peers globally [4]. The same finding was reported by Lucia et al. 2020 in the USA among medical students [38].

On comparing the vaccine-related attitudes among HCS in the USA, Kelekar et al. 2021 found that medical students (23%) had significantly lower levels of COVID-19 vaccine hesitancy than dental students (45%) [60]. In recent cross-sectional studies, medical students had higher levels of hepatitis-B virus (HBV) vaccine coverage in Greece (88.1% vs. 81.4%) and France (92% vs. 88%) than nursing students [61,62]. Therefore, our study is limited because all medical and non-medical healthcare students were pooled in one group, thus overlooking the differences between healthcare study programs.

Vecchia et al. 2020 found that lack of intentions to receive influenza vaccine and high levels of COVID-19 vaccine hesitancy among the Italian adult population were both expressed by less qualified workers [63]. Last summer, Gostin et al. 2020 called upon health systems globally to be prepared for the co-epidemics of COVID-19 and influenza that require expansion of influenza vaccine coverage [64]. While there is a lack of evidence supporting Gostin’s recommendation’s effectiveness, we found that the students who received the influenza vaccine were 1.510 times (*CI 95%:* 1.099–2.067; *Sig.* = 0.011) more likely to accept the COVID-19 vaccine. Our findings may justify the multi-antigen vaccination campaigns, not only in low-income settings but also in high- and middle-income settings, especially in the context of respiratory infectious diseases to relieve the pressure on our weakened healthcare systems in the post-COVID-19 era.

Previous COVID-19 infection decreased the Czech university students’ acceptance level of the COVID-19 vaccine, which might be caused by a false sense of protection against the virus. The participants who had been infected by COVID-19 (30.3%) had a significantly higher level of immunity misconception (*χ^2^* = 11.023; *Sig.* = 0.001) compared to the participants who were not infected previously (21.8%). A recent study demonstrated high levels of vaccine hesitancy (59.2%) among Italian patients who recovered from COVID-19, which was associated with younger age and a milder clinical course of the infection [65]. In the USA, recovered COVID-19 patients were mainly resistant (54%) to getting vaccinated, with the cardiac and diabetic patients being more likely to reject the vaccine [66].

The time-series analysis revealed that vaccine acceptance level was declining steadily over the weeks of our study, 74.7% in the first week vs. 59% in the last week. The first explanation for this decline is the high proportion of HCS during the first weeks of the study that was not maintained in the last weeks. This proposition might not be accurate because the subgroup analysis showed that this declining trend was observed among HCS and non-HCS. The second explanation is that a considerable portion of the university students were already vaccinated by the time the survey was sent to them, especially in the later weeks, simply because they were engaged with voluntary health and social care activities and frontline duties. The third explanation is attributed to the emerging news about variants of SARS-CoV-2 that may escape the current vaccines and decrease the effectiveness of mass vaccination efforts [67]. On 7 April 2021, the former Czech minister of health, Jan Blatny, was dismissed from his post due to disputes over the Sputnik V vaccine which the Czech prime minister incentivized to enter the Czech market even without the approval of the European Medicines Agency (EMA) [68]. This incident, and other similar incidents, are believed to have had impacted public confidence in COVID-19 vaccines and the whole vaccination strategies. Although this finding is limited, it shows that vaccine attitudes are most likely not stable, and they are probably sensitive to public opinions and events.

In total, 81.3% of the participating students reported that their vaccination decision was not influenced by the news they heard from mass media or social media. However, this self-reported finding does not fully correspond with the time-series analysis discussed previously; it is worth mentioning that fighting misinformation and raising digital literacy are the core objectives of several non-governmental organizations and initiatives launched recently in the Czech Republic [69,70,71]. The COVID-19-associated infodemic is a public health threat that was warned against persistently by the WHO; therefore, transmitting timely and high-quality information was an essential tool used by the Chinese government to control the acute phase of the pandemic [72]. Recent discourse analysis revealed that COVID-19 vaccine hesitancy tweets were associated with safety issues, mistrust of governments and the pharmaceutical industry, and insufficient knowledge about the vaccines [73]. This anti-vaccine content in Twitter increased significantly during the first half of 2020 [74].

One-quarter of the participants (25.5%) reported that their vaccination decision would be influenced by the vaccine side effects being monitored closely by competent authorities. A recent Polish study demonstrated that passive surveillance systems used widely by the governments to monitor COVID-19 vaccine side effects were ineffective, thus bolding the demand for active surveillance of COVID-19 vaccine side effects by independent (non-sponsored) studies [75,76,77,78,79,80,81]. The students who reported to need this evidence the most are mainly females, non-HCS, influenced by media and social media, mistrusting their healthcare providers and pharmaceutical industry, and with immunity misconception and insufficient perceived knowledge.

### 4.1. Strengths

To the best of the authors’ knowledge, this is the first study to evaluate the COVID-19 vaccine hesitancy in the Czech Republic and investigate the potential drivers of this demanding public health challenge. The target population of this study, university students, represent a unique subset of the general population that retains the highest possible levels of health literacy and health-related knowledge, and they are perceived as the opinion leaders within their communities; therefore, their health-related beliefs and attitudes affect to a considerable extent their general population. This study managed to shed light on the differences across gender, study fields, and academic years regarding COVID-19 vaccine hesitancy and its drivers, which are deemed required to synthesize evidence-informed policy recommendations.

### 4.2. Limitations

The first limitation of this study is due to the snowballing technique that made some groups underrepresented, such as SGM, non-Czech students, and non-HCS. The second limitation is due to following the methodology of the Czech Statistical Office (ČSÚ) for the study fields categorization that is adapted from the International Standard Classification of Education Fields (ISCED-F 2013) of the United Nations Educational, Scientific and Cultural Organization (UNESCO), because it was unable to track the differences between medical (e.g., general medicine and dentistry) and non-medical (e.g., nursing, midwifery, and paramedic practice) healthcare programs [52,82].

### 4.3. Implications

This study calls for promotional interventions in the Czech universities to target non-HCS as they were found to retain lower levels of vaccine acceptance and vaccine-related awareness. Our findings warrant designing educational programs for high school students in the Czech Republic to increase their understanding of vaccines and immunization as most of those students will not pursue healthcare-related studies, while they will still be responsible for vaccinating their children in the future. The primary prevention dialogue in the Czech Republic needs to be culturally sensitive and inclusive for all foreign nationals living in the Republic. Future studies of university students’ vaccine-related attitudes should discriminate between medical and non-medical healthcare students. The different healthcare study programs may impact the students’ beliefs and attitudes towards preventive medicine differently. The findings of this study can be utilized to establish a health belief model to predict students’ attitudes towards vaccination in the Czech Republic.

## 5. Conclusions

The Czech university students demonstrated a substantial level of COVID-19 vaccine acceptance (73.3%), a low level of vaccine resistance (19.3%), and a very low level of vaccine hesitancy (7.4%) that predict a fair probability to achieve community immunity (herd immunity) among this population group. Vaccine promotional campaigns should target non-HCS as they were more reluctant to accept COVID-19 vaccination. The primary prevention strategies in the Czech Republic need to be culturally sensitive and inclusive for foreign nationals. As one-quarter of the participating students are dependent on vaccine safety data, the findings of this study support the call for independent studies evaluating the side effects of COVID-19 vaccines.

## Figures and Tables

**Table 1:** Demographic characteristics of participating Czech university students (April–June 2021, *n* = 1351).

Variable	Outcome	Frequency	Percentage
Gender	Female	903	66.8%
	Male	439	32.5%
	Prefer not to say	9	0.7%
Age	≤22 years-old	726	53.7%
	>22 years-old	625	46.3%
Nationality	Czech	1142	84.5%
	Slovak	187	13.8%
	Russian	9	0.7%
	Ukrainian	8	0.6%
	Kazakh	2	0.1%
	Belarusian	1	0.1%
	Bosnian	1	0.1%
	Vietnamese	1	0.1%
University	Masaryk University	452	33.5%
	Charles University	276	20.4%
	Brno University of Technology	212	15.7%
	Palacky University Olomouc	92	6.8%
	Mendel University in Brno	81	6%
	University of Defence	57	4.2%
	Janáček Academy of Music in Brno	57	4.2%
	Czech University of Life Sciences in Prague	38	2.8%
	Silesian University in Opava	22	1.6%
	Prague University of Economics and Business	20	1.5%
	Technical University of Ostrava	18	1.3%
	University of Chemistry and Technology in Prague	4	0.3%
	Tomas Bata University in Zlín	4	0.3%
	Czech Technical University in Prague	3	0.2%
	University of Ostrava	3	0.2%
	Other	12	0.9%
Field of Study	Medical and Healthcare Sciences	549	40.6%
	Technical Sciences	200	14.8%
	Social Sciences	125	9.3%
	Business and Economics	105	7.8%
	Natural Sciences	104	7.7%
	Education and Social Care	102	7.5%
	Agriculture, Forestry and Veterinary Sciences	54	4%
	Arts and Humanities	52	3.8%
	Military Sciences	33	2.4%
	Law	13	1%
	Not Specified	14	1%
Medical and Healthcare Faculties	Faculty of Medicine, Masaryk University	229	41.7%
	1st Faculty of Medicine, Charles University	1	0.2%
	2nd Faculty of Medicine, Charles University	154	28.1%
	Faculty of Medicine in Hradec Kralove, Charles University	117	21.3%
	Faculty of Medicine, University of Ostrava	1	0.2%
	Faculty of Medicine, Palacky University Olomouc	20	3.6%
	Faculty of Pharmacy, Masaryk University	25	4.6%
	Faculty of Pharmacy, Charles University	2	0.4%
Academic Level	1st Year	327	24.2%
	2nd Year	265	19.6%
	3rd Year	248	18.4%
	4th Year (1st Year of Follow-up Masters)	165	12.2%
	5th Year (2nd Year of Follow-up Masters)	191	14.1%
	6th Year	78	5.8%
	Doctoral Candidate	77	5.7%

**Table 2:** COVD-19-related anamnesis and influenza vaccine experience of participating Czech university students (April–June 2021, *n* = 1351).

Outcome	Female	Male	*Sig.*	Czech	Non-Czech	*Sig.*	HCS	Non-HCS	*Sig.*	Total
COVID-19 Infection	257 (28.5%)	141 (32.1%)	0.169	347 (30.4%)	53 (25.4%)	0.143	169 (30.8%)	231 (28.8%)	0.434	400 (29.6%)
Providing Care	303 (33.6%)	135 (30.8%)	0.304	387 (33.9%)	55 (26.3%)	0.032	211 (38.4%)	231 (28.8%)	<0.001	442 (32.7%)
Knowing Patient	893 (98.9%)	434 (98.9%)	0.959	1128 (98.8%)	207 (99%)	0.741	542 (98.7%)	793 (98.9%)	0.799	1335 (98.8%)
Knowing Dead	329 (36.4%)	141 (32.1%)	0.120	404 (35.4%)	68 (32.5%)	0.428	188 (34.2%)	284 (35.4%)	0.658	472 (34.9%)
Flu Vaccine	177 (19.6%)	100 (22.8%)	0.177	214 (18.7%)	66 (31.6%)	<0.001	133 (24.2%)	147 (18.3%)	0.009	280 (20.7%)
*Recent Flu Vaccine*	78 (44.6%)	46 (46%)	0.819	98 (46%)	27 (41.5%)	0.526	84 (63.6%)	41 (28.1%)	<0.001	125 (45%)

HCS = Healthcare Students. Non-HCS = Non-Healthcare Students. Chi-squared test (χ^2^) was used with a significance level (*Sig.*) of ≤0.05.

**Table 3:** COVID-19 vaccine-related attitudes of participating Czech university students (April–June 2021, *n* = 1351).

Outcome	Female	Male	*Sig.*	Czech	Non-Czech	*Sig.*	HCS	Non-HCS	*Sig.*	Total
Strongly Disagree (1)	84 (9.3%)	53 (12.1%)	0.116	122 (10.7%)	17 (8.1%)	0.265	42 (7.7%)	97 (12.1%)	0.008	139 (10.3%)
Disagree (2)	84 (9.3%)	37 (8.4%)	0.600	107 (9.4%)	15 (7.2%)	0.309	38 (6.9%)	84 (10.5%)	0.025	122 (9%)
Not Sure (3)	75 (8.3%)	23 (5.2%)	0.043	90 (7.9%)	10 (4.8%)	0.116	30 (5.5%)	70 (8.7%)	0.024	100 (7.4%)
Agree (4)	181 (20%)	92 (21%)	0.697	234 (20.5%)	39 (18.7%)	0.545	94 (17.1%)	179 (22.3%)	0.019	273 (20.2%)
Totally Agree (5)	479 (53%)	234 (53.3%)	0.929	589 (51.6%)	128 (61.2%)	0.010	345 (62.8%)	372 (46.4%)	<0.001	717 (53.1%)
Total (1–5)	3.98 ± 1.35	3.95 ± 1.42	0.915	3.93 ± 1.39	4.18 ± 1.29	0.008	4.21 ± 1.27	3.80 ± 1.42	<0.001	3.97 ± 1.38

HCS = Healthcare Students. Non-HCS = Non-Healthcare Students. Chi-squared test (χ^2^) and Mann-Whitney (*U*) used with a significance level (*Sig.*) of ≤0.05.

**Table 4:** COVID-19 vaccine-related attitude drivers of participating Czech university students stratified by gender, nationality, and field (April–June 2021, *n* = 1351).

Variable	Outcome	Female	Male	*Sig.*	Czech	Non-Czech	*Sig.*	HCS	Non-HCS	*Sig*.	Total
**Contextual Drivers**											
Media and Social Media	No (0)	717 (79.4%)	379 (86.3%)	0.002	916 (80.2%)	182 (87.1%)	0.019	454 (82.7%)	644 (80.3%)	0.287	1098 (81.3%)
	Not Sure (1)	63 (7%)	21 (4.8%)	0.120	79 (6.9%)	6 (2.9%)	0.027	31 (5.6%)	54 (6.7%)	0.428	85 (6.3%)
	Yes (2)	123 (13.6%)	39 (8.9%)	0.012	147 (12.9%)	21 (10%)	0.255	64 (11.7%)	104 (13%)	0.474	168 (12.4%)
Personal Beliefs	No (0)	777 (86%)	399 (90.9%)	0.011	999 (87.5%)	182 (87.1%)	0.874	493 (89.8%)	688 (85.8%)	0.029	1181 (87.4%)
	Not Sure (1)	23 (2.5%)	2 (0.5%)	0.008	22 (1.9%)	4 (1.9%)	0.990	10 (1.8%)	16 (2%)	0.820	26 (1.9%)
	Yes (2)	103 (11.4%)	38 (8.7%)	0.123	121 (10.6%)	23 (11%)	0.860	46 (8.4%)	98 (12.2%)	0.025	144 (10.7%)
Pharmaceutical Industry	No (0)	109 (12.1%)	63 (14.4%)	0.241	162 (14.2%)	14 (6.7%)	0.003	39 (7.1%)	137 (17.1%)	<0.001	176 (13%)
	Not Sure (1)	123 (13.6%)	41 (9.3%)	0.025	148 (13%)	18 (8.6%)	0.078	53 (9.7%)	113 (14.1%)	0.015	166 (12.3%)
	Yes (2)	671 (74.3%)	335 (76.3%)	0.427	832 (72.9%)	177 (84.7%)	<0.001	457 (83.2%)	552 (68.8%)	<0.001	1009 (74.7%)
**Social Drivers**											
Health System	No (0)	185 (20.5%)	82 (18.7%)	0.436	245 (21.5%)	25 (12.4%)	0.003	79 (14.4%)	192 (23.9%)	<0.001	271 (20.1%)
	Not Sure (1)	164 (18.2%)	71 (16.2%)	0.369	197 (17.3%)	40 (19%)	0.509	83 (15.1%)	154 (19.2%)	0.053	237 (17.5%)
	Yes (2)	554 (61.4%)	286 (65.1%)	0.177	700 (61.3%)	143 (68.4%)	0.051	387 (70.5%)	456 (56.9%)	<0.001	843 (62.4%)
Perceived Knowledge	No (0)	289 (32%)	115 (26.2%)	0.030	359 (31.4%)	46 (22%)	0.006	123 (22.4%)	282 (35.2%)	<0.001	405 (30%)
	Not Sure (1)	104 (11.5%)	57 (13%)	0.438	135 (11.8%)	28 (13.4%)	0.520	66 (12%)	97 (12.1%)	0.968	163 (12.1%)
	Yes (2)	510 (56.5%)	267 (60.8%)	0.131	648 (56.7%)	135 (64.6%)	0.035	360 (65.6%)	423 (52.7%)	<0.001	783 (58%)
Immunity Misconception	No (0)	474 (52.5%)	246 (56%)	0.222	578 (50.6%)	145 (69.4%)	<0.001	363 (66.1%)	360 (44.9%)	<0.001	723 (53.5%)
	Not Sure (1)	214 (23.7%)	82 (18.7%)	0.037	265 (23.2%)	35 (16.7%)	0.039	86 (15.7%)	214 (26.7%)	<0.001	300 (22.2%)
	Yes (2)	215 (23.8%)	111 (25.3%)	0.554	299 (26.2%)	29 (13.9%)	<0.001	100 (18.2%)	228 (28.4%)	<0.001	328 (24.3%)
**Vaccine-specific Drivers**											
Novel Vaccines	No (0)	597 (66.1%)	290 (66.1%)	0.984	730 (63.9%)	161 (77%)	<0.001	403 (73.4%)	488 (60.8%)	<0.001	891 (66%)
	Not Sure (1)	117 (13%)	49 (11.2%)	0.349	153 (13.4%)	16 (7.7%)	0.021	48 (8.7%)	121 (15.1%)	0.001	169 (12.5%)
	Yes (2)	189 (20.9%)	100 (22.8%)	0.440	259 (22.7%)	32 (15.3%)	0.017	98 (17.9%)	193 (24.1%)	0.006	291 (21.5%)
Safety Surveillance	No (0)	480 (53.2%)	301 (68.6%)	<0.001	649 (56.8%)	135 (64.6%)	0.037	343 (62.5%)	441 (55%)	0.006	784 (58%)
	Not Sure (1)	173 (19.2%)	47 (10.7%)	<0.001	196 (17.2%)	26 (12.4%)	0.090	84 (15.3%)	138 (17.2%)	0.353	222 (16.4%)
	Yes (2)	250 (27.7%)	91 (20.7%)	0.006	297 (26%)	48 (23%)	0.354	122 (22.2%)	223 (27.8%)	0.021	345 (25.5%)
Local Availability	No (0)	422 (46.7%)	207 (47.2%)	0.885	555 (48.6%)	81 (38.8%)	0.009	265 (48.3%)	371 (46.3%)	0.467	636 (47.1%)
	Not Sure (1)	274 (30.3%)	130 (29.6%)	0.784	327 (28.6%)	78 (37.3%)	0.012	169 (30.8%)	236 (29.4%)	0.593	405 (30%)
	Yes (2)	207 (22.9%)	102 (23.2%)	0.899	260 (22.8%)	50 (23.9%)	0.715	115 (20.9%)	195 (24.3%)	0.148	310 (22.9%)

HCS = Healthcare Students. Non-HCS = Non-Healthcare Students. Chi-squared test (χ^2^) was used with a significance level (*Sig.*) of ≤0.05.

**Table 5:** Demographic and anamnestic determinants of Czech university students’ COVID-19 vaccine acceptance (April–June 2021, *n* = 1351).

Variable	Outcome	*n* (%)	Acceptance Level	*Sig.*
Gender	Female	903 (66.8%)	3.98 ± 1.35	0.558
	Male	439 (32.5%)	3.95 ± 1.42	
	Prefer not to say	9 (0.7%)	3.33 ± 1.73	
Age	≤22 years-old	726 (53.7%)	4.02 ± 1.32	0.473
	>22 years-old	625 (46.3%)	3.91 ± 1.44	
Nationality	Czech	1142 (84.5%)	3.93 ± 1.39	0.066
	Slovak	187 (13.8%)	4.17 ± 1.30	
	Russian	9 (0.7%)	3.67 ± 1.23	
	Ukrainian	8 (0.6%)	4.38 ± 1.41	
	Kazakh	2 (0.1%)	5 ± 0	
	Belarusian	1 (0.1%)	5	
	Bosnian	1 (0.1%)	5	
	Vietnamese	1 (0.1%)	5	
University	Masaryk University	452 (33.5%)	4 ± 1.34	<0.001
	Charles University	276 (20.4%)	4.36 ± 1.15	
	Brno University of Technology	212 (15.7%)	4.01 ± 1.37	
	Palacky University Olomouc	92 (6.8%)	3.87 ± 1.45	
	Mendel University in Brno	81 (6%)	3.56 ± 1.50	
	University of Defence	57 (4.2%)	3.81 ± 1.33	
	Janáček Academy of Music in Brno	57 (%4.2)	3.40 ± 1.51	
	Czech University of Life Sciences in Prague	38 (2.8%)	3.21 ± 1.55	
	Silesian University in Opava	22 (1.6%)	3.41 ± 1.47	
	Prague University of Economics and Business	20 (1.5%)	4.45 ± 1.23	
	Technical University of Ostrava	18 (1.3%)	3.06 ± 1.63	
	University of Chemistry and Technology in Prague	4 (0.3%)	5	
	Tomas Bata University in Zlín	4 (0.3%)	4.5 ± 1	
	Czech Technical University in Prague	3 (0.2%)	3.67 ± 2.31	
	University of Ostrava	3 (0.2%)	3.67 ± 1.53	
Field of Study	Medical and Healthcare Sciences	549 (40.6%)	4.21 ± 1.27	<0.001
	Technical Sciences	200 (14.8%)	4.03 ± 1.36	
	Social Sciences	125 (9.3%)	3.94 ± 1.26	
	Business and Economics	105 (7.8%)	3.97 ± 1.39	
	Natural Sciences	104 (7.7%)	3.86 ± 1.42	
	Education and Social Care	102 (7.5%)	3.61 ± 1.48	
	Agriculture, Forestry and Veterinary Sciences	54 (4%)	3.20 ± 1.53	
	Arts and Humanities	52 (3.8%)	3.38 ± 1.64	
	Military Sciences	33 (2.4%)	3.73 ± 1.18	
	Law	13 (1%)	3.85 ± 1.68	
Medical and Healthcare Faculties	Faculty of Medicine, Masaryk University	229 (41.7%)	4.05 ± 1.34	0.001
	1st Faculty of Medicine, Charles University	1 (0.2%)	5	
	2nd Faculty of Medicine, Charles University	154 (28.%)	4.52 ± 1.09	
	Faculty of Medicine in Hradec Kralove, Charles University	117 (21.3%)	4.13 ± 1.25	
	Faculty of Medicine, University of Ostrava	1 (0.2%)	2	
	Faculty of Medicine, Palacky University Olomouc	20 (3.6%)	3.85 ± 1.66	
	Faculty of Pharmacy, Masaryk University	25 (4.6%)	4.36 ± 1.04	
	Faculty of Pharmacy, Charles University	2 (0.4%)	5	
Academic Year	1st Year	327 (24.2%)	3.97 ± 1.34	0.343
	2nd Year	265 (19.6%)	3.97 ± 1.34	
	3rd Year	248 (18.4%)	3.99 ± 1.38	
	4th Year (1st Year of Follow-up Masters)	165 (12.2%)	3.87 ± 1.43	
	5th Year (2nd Year of Follow-up Masters)	191 (14.1%)	3.90 ± 1.41	
	6th Year	78 (5.8%)	4.24 ± 1.34	
	Doctoral Student	77 (5.7%)	3.97 ± 1.45	

Mann–Whitney (*U*) and Kruskal–Wallis (*H*) tests were used with a significance level (*Sig.*) of ≤0.05.

**Table 6:** COVID-19 vaccine-related attitudes of Czech university students by week (April–June 2021, *n* = 1351).

Week	Date	Vaccine Resistant	Vaccine Hesitant	Vaccine Accepting	Acceptance Level
1st Week	21–27 April 2021	84 (18.7%)	30 (6.7%)	336 (74.7%)	4.01 ± 1.38
2nd Week	28 April–4 May 2021	116 (18.3%)	42 (6.6%)	475 (75%)	4.03 ± 1.35
3rd Week	5–11 May 2021	13 (17.8%)	9 (12.3%)	51 (69.9%)	3.86 ± 1.26
4th Week	2–8 June 2021	30 (22.4%)	12 (9%)	92 (68.7%)	3.83 ± 1.46
5th Week	9–15 June 2021	18 (29.5%)	7 (11.5%)	36 (59%)	3.39 ± 1.43
*Sig.*		0.239	0.246	0.046	<0.001

Chi-squared test (χ^2^) and Kruskal–Wallis (*H*) tests were used with a significance level (*Sig.*) of ≤0.05.

**Table 7:** Logistic regression of Czech university students’ COVID-19 vaccine attitude determinants and drivers (April–June 2021, *n* = 1351).

	Variable	Vaccine Hesitancy			Variable	Vaccine Acceptance		
		B (SE)	OR	CI 95%		B (SE)	OR	CI 95%
Demographic Determinants	Female (vs. Male)	0.494 (0.246)	1.638	1.012–2.652	Male (vs. Female)	0.060 (0.132)	1.062	0.819–1.377
	Gender: SGM (vs. >Declared)	1.288 (0.809)	3.627	0.743–17.694	*Gender*: Declared (vs. >SGM)	1.242 (0.674)	3.462	0.925–12.965
	≤22 years (vs. >22 years)	0.601 (0.220)	1.824	1.185–2.808	>22 years (vs. ≤22 years)	0.076 (0.123)	1.079	0.847–1.373
	Czech (vs. Non-Czech)	0.532 (0.342)	1.702	0.871–3.329	Non-Czech (vs. Czech)	0.433 (0.185)	1.541	1.073–2.214
	Non-HCS (vs. HCS)	0.503 (0.226)	1.654	1.063–2.575	HCS (vs. Non-HCS)	0.598 (0.131)	1.818	1.406–2.350
	*HCS*: PMBD (vs. CMMD)	1.020 (0.547)	2.774	0.950–8.103	*HCS*: CMMD (vs. PMBD)	0.284 (0.245)	1.329	0.821–2.150
Anamnestic Determinants	No Infection (vs. Infection)	−0.501 (0.213)	0.606	0.399–0.921	Infection (vs. no Infection)	−0.128 (0.133)	0.880	0.678–1.143
	Not Providing Care (vs. Care)	0.085 (0.224)	1.089	0.701–1.691	Providing Care (vs. no Care)	0.0.36 (0.131)	1.037	0.801–1.342
	Not Knowing Patient (vs. Patient)	*NV*	*NV*	*NV*	Knowing Patient (vs. no Patient)	0.768 (0.508)	2.155	0.797–5.831
	Not Knowing Dead (vs. Dead)	0.455 (0.235)	1.577	0.994–2.502	Knowing Dead (vs. no Dead)	0.327 (0.133)	1.386	1.069–1.798
	No Flu Vaccine (vs. Flu Vaccine)	0.506 (0.296)	1.659	0.928–2.965	Flu Vaccine (vs. No Flu Vaccine)	0.412 (0.162)	1.510	1.099–2.076
	Flu Vaccine: No Recent (vs. Recent)	0.637 (0.613)	1.891	0.568–6.292	*Flu Vaccine*: Recent (vs. no Recent)	0.146 (0.300)	1.157	0.642–2.085
Vaccine-related Attitudes Drivers	Media/Social Media: Yes (vs. No)	1.127 (0.240)	3.086	1.928–4.941	*Media/Social Media*: No (vs. Yes)	0.566 (0.148)	1.761	1.317–2.355
	Personal Beliefs: Yes (vs. No)	0.428 (0.295)	1.535	0.861–2.737	*Personal Beliefs*: No (vs. Yes)	1.617 (0.171)	5.036	3.602–7.039
	Pharma. Industry: No (vs. Yes)	0.826 (0.250)	2.283	1.400–3.724	*Pharma. Industry*: Yes (vs. No)	2.470 (0.146)	11.828	8.886–15.742
	Health System: No (vs. Yes)	0.889 (0.221)	2.432	1.578–3.747	*Health System*: Yes (vs. No)	1.711 (0.133)	5.535	4.265–7.183
	Perceived Knowledge: No (vs. Yes)	1.802 (0.225)	6.061	3.897–9.429	*Perceived Knowledge*: Yes (vs. No)	1.187 (0.128)	3.277	2.550–4.212
	Misconception: Yes (vs. No)	1.208 (0.211)	3.347	2.212–5.064	*Misconception*: No (vs. Yes)	1.581 (0.137)	4.857	3.717–6.348
	Novel Vaccines: Yes (vs. No)	0.589 (0.225)	1.802	1.159–2.804	*Novel Vaccines*: No (vs. Yes)	1.584 (0.131)	4.876	3.772–6.302
	Safety Surveillance: Yes (vs. No)	1.087 (0.211)	2.965	1.961–4.483	*Safety Surveillance*: No (vs. Yes)	0.499 (0.124)	1.648	1.292–2.100
	Local Availability: No (vs. Yes)	0.040 (0.208)	1.041	0.692–1.565	*Local Availability*: Yes (vs. No)	0.309 (0.153)	1.362	1.009–1.838

SGM = Sexual and Gender Minority. HCS = Healthcare Students. Non-HCS = Non-Healthcare Students.

## Data Availability

The data that support the findings of this study are available from the corresponding author upon reasonable request.

## Supplementary Materials

The following are available online at https://www.mdpi.com/article/10.3390/vaccines9090948/s1, Table S1. The results of the test re-test reliability, Table S2. Vaccine hesitancy drivers adapted from the WHO-SAGE compendium, Table S3. Distribution of Participating Czech Universities Students by Week (April– June 2021, *n* = 1351).
