# Supplementary material for: Prevalence and Drivers of COVID-19 Vaccine Hesitancy among Czech University Students: National Cross-Sectional Study
Source: Vaccines (Basel). 2021 Aug 25;9(9):948. doi: 10.3390/vaccines9090948 (PMC8470400; doi:10.3390/vaccines9090948)
Supplement: Supplementary file 1 [file vaccines-09-00948-s001.zip › vaccines-1305497-supplementary.pdf]

Table S1. The results of the test re-test reliability

Participant	κ Coefficient	Participant	κ Coefficient
No. 1	0.931	No. 6	0.797
No. 2	0.931	No. 7	0.935
No. 3	0.595	No. 8	0.519
No. 4	0.931	No. 9	1.000
No. 5	0.793	Total	0.83 ± 0.17

Cohen's Kappa statistic (κ): 0.01–0.2 as none to slight, 0.21–0.4 as fair, 0.41–0.6 as moderate, 0.61–0.80 as substantial, and 0.81–1.0 as perfect agreement.[1]

Table S2. Vaccine hesitancy drivers adapted from the WHO-SAGE compendium.[2]

**Item**	*Answers*
**Contextual Drivers**	
**1)** Have reports you heard/read in the media/ on social media made you re-consider the choice to take COVID-19 vaccine?	Yes/Not Sure/No
**2)** Have you refused taking a drug or a vaccine due to your cultural, religious, or personal values?	Yes/Not Sure/No
**3)** Do you trust pharmaceutical companies to provide safe and effective vaccines?	Yes/Not Sure/No
**Social Drivers**	
**1)** Do you trust your health care provider to tell you about the risks and benefits of vaccines honestly?	Yes/Not Sure/No
**2)** Do you feel you have enough information about vaccines and their safety?	Yes/Not Sure/No
**3)** Do you believe that it is better to develop immunity by getting sick that to get a vaccine shot?	Yes/Not Sure/No
**Vaccine-specific Drivers**	
**1)** Do you think that new vaccines are not trailed to the same rigorous standard as any normally prescribed drug?	Yes/Not Sure/No
**2)** If the vaccine side effects and adverse reactions are kept track, would that affect your decision regarding taking the vaccine?	Yes/Not Sure/No
**3)** Do you feel confident that the health centre or doctor's office will have the vaccine you need, when you need them?	Yes/Not Sure/No

Table S3. Distribution of Participating Czech Universities Students by Week (April – June 2021, n = 1351)

Week	Date	Participants	Female	Czech	HCS
1st Week	April 21st – April 27th, 2021	450 (33.3%)	269 (59.8%)	359 (79.8%)	101 (22.4%)
2nd Week	April 28th – May 4th, 2021	633 (46.9%)	454 (71.7%)	529 (83.6%)	433 (68.4%)
3rd Week	May 5th – May 11^{th,} 2021	73 (5.4%)	31 (42.5%)	68 (93.2%)	15 (20.5%)
4th Week	June 2nd – June 8th, 2021	134 (9.9%)	108 (80.6%)	128 (95.5%)	0 (0%)
5th Week	June 9th – June 15th, 2021	61 (4.5%)	41 (67.2%)	58 (95.1%)	0 (0%)

References

1. McHugh ML. Interrater reliability: The kappa statistic. *Biochem Medica*. 2012;22(3):276-282. doi:10.11613/bm.2012.031
2. Strategic Advisory Group of Experts on Immunization (SAGE). *Vaccine Hesitancy Survey Questions Related to SAGE Vaccine Hesitancy Matrix*. Accessed March 14, 2021. https://www.who.int/immunization/programmes_systems/Survey_Questions_Hesitancy.pdf
